# Collaborative leadership in team science: dynamics of sense making, decision making, and action taking

**DOI:** 10.3389/frma.2023.1211407

**Published:** 2023-06-21

**Authors:** Gemma Jiang

**Affiliations:** Institute for Research in the Social Sciences, Colorado State University, Fort Collins, CO, United States

**Keywords:** collaborative leadership, team science, complexity, Theory U, Divergence-Convergence Diamond, Strategic Doing

## Abstract

To help cross-disciplinary science teams navigate through internal and external complexities, this perspective article explores the application of three conceptual frameworks: Theory U, Divergence-Convergence Diamond, and Strategic Doing. These frameworks enable science teams to avoid common pitfalls by operationalizing collaborative leadership as iterative cycles of distributed sense making, decision making and action taking. Implications for team science practices include facilitating the process, prototyping the future and applying dynamic roles and responsibilities.

## 1. Introduction

Cross-disciplinary science teams are embedded in high degrees of internal and external complexities. In this perspective article, we refer to a specific type of science teams that are formed in response to federal funding initiatives to address pressing scientific and societal challenges, that span several disciplines and higher education institutions, and typically last 3–5 years.

Internally, such teams face challenges such as high diversity of membership, deep knowledge integration, large size, goal misalignment among team members, and high task interdependence (National Research Council, 2015). Externally, such teams need to work with different institutional constraints, and more broadly complex political, technological, environmental landscapes, and changing science funding priorities. To be successful in navigating through the complexities, it is helpful to conceptualize cross-disciplinary science teams as complex adaptive systems that seek emergent outcomes such as learning, innovation, and adaptability, as opposed to standardization, predictability and repetition of known processes. In complex adaptive systems, interactions among interdependent agents, and with their contexts lead to nonlinear and emergent changes (Cilliers, 2002; Uhl-Bien et al., 2007; Johnson, 2012). The direction of change cannot be predicted but can be influenced. A central leadership question becomes *how to influence change toward desired outcomes*.

In this perspective article, we operationalize leadership as iterative cycles of distributed sense making, decision making and action taking. The universal process of “thinking, deciding, acting” can happen in a split second for unconscious processes such as stopping at a traffic light. Much more deliberate processes are needed for complex science teams navigating through their ever more complex and ambiguous contexts. It is self-evident that successful leadership depends on effective *decision making* and *action taking*. However, the third vital component - *sense making*- is typically given much less attention and is often overlooked completely. That becomes problematic, because how teams make sense of things influences the decisions they make and the actions they take, as discussed in the sensemaking literature (Weick, 1979, 1995; Weick et al., 2005). In an increasingly uncertain and unpredictable world, *sense making, decision making, and action taking* must become ever more rapidly and repeatedly iterated, deeply embedded, and widely distributed throughout the teams (Marlow, 2023).

## 2. Challenges

Most science teams aspire to practice collaborative leadership, which calls for distributed sense making, decision making and action taking. However, the long history of top down autocratic leadership practices implicitly assumes that decisions are made by the most senior people. This has become so axiomatic that the terms “senior person” and “decision maker” are typically treated as synonymous. This creates a huge capacity building need for cross-disciplinary science teams, since most of them are embedded in this default centralized decision-making tradition prevalent in most higher education institutes.

Complexity leadership, the theoretical foundation for this perspective article, assumes that the interactive dynamics among interdependent actors are responsible for outcomes. Leadership therefore is distributed, collective, collaborative and shared across relevant actors in the system (Uhl-Bien et al., 2007; Arena and Uhl-Bien, 2016; Uhl-Bien and Arena, 2017; Uhl-Bien, 2021). This theoretical foundation has been successfully applied in many different contexts, such as business innovation (Schoemaker et al., 2018), team effectiveness (Wang et al., 2014), management (Alvesson and Willmott, 2012), and team science (Jiang et al., 2023).

From my experience working as a team scientist across many different National Science Foundation funded programs, I have identified three pitfalls most science teams are prone to in practicing collaborative leadership.

### 2.1. Pitfall #1: perpetual sense making with no decisions made and no actions taken

The biggest complaint I hear is “Meeting after meeting, we talk about things, but no decision is ever made. I would like to see some action, some progress.” This seems to be a particular pertinent challenge for scientists because they are trained to be analytical. However, too much analysis can leave the team stuck in “analysis paralysis” and lose momentum for important initiatives. Despite the stated intention to explore the frontier of knowledge, certain aspects of science culture are risk averse. Culturally conditioned to avoid failure, scientists tend to bias toward not taking action until they know it's the right action - by which time, in a volatile, uncertain, complex and ambiguous world, it's no longer the right action.

### 2.2. Pitfall #2: decisions are made without adequately inclusive sensemaking

When this happens, I often hear the following comments from team members, “It seems to me the leaders have already made up their minds. I do not think there is any room for our input.” There are usually two consequences for this pitfall: (1) the decision is not optimal because important information is missing from lack of thorough sensemaking; (2) action taking falls apart or runs into strong resistance from lack of buy-in from team members for whom the decision doesn't make sense.

### 2.3. Pitfall #3: no adaptation in action plans to changing contexts

This quote is a good illustration of this pitfall: “The project management plan is our bible. Everyone just needs to read the plan and will know exactly what to do.” Teams are most likely to fall into this pitfall as they navigate the team forming stage shortly after funding. As the military saying goes, “no plan survives first contact with the enemy”. The “enemy” in this context could be the distance between knowing what to do, and actually doing it, which in essence is the distance between the two-dimensional world of thoughts during the proposal stage and the three-dimensional world of reality during the implementation stage. The “enemy” could also be the actuality of the situation as opposed to the assumed reality of the situation when the plan was made. Without the iterative sense making, decision making, and action taking loop, teams usually face two bigger pitfalls down the road: (1) some team members are disengaged because they were not part of the proposal writing process; (2) teams get stuck because the plan is not serving the changed reality.

## 3. Frameworks

We explore the application of three conceptual frameworks that have proven effective in helping teams avoid the pitfalls above. The conceptual frameworks, Theory U (Senge et al., 2008; Scharmer, 2009, 2018), Divergence-Convergence Diamond (Kaner, 2014), and Strategic Doing (Morrison et al., 2019), each with their distinctive intellectual lineages and practical applications, support collaborative leadership operationalized as iterative cycles of distributed sense making, decision making, and action taking.

### 3.1. Theory U

**Figure 1:**
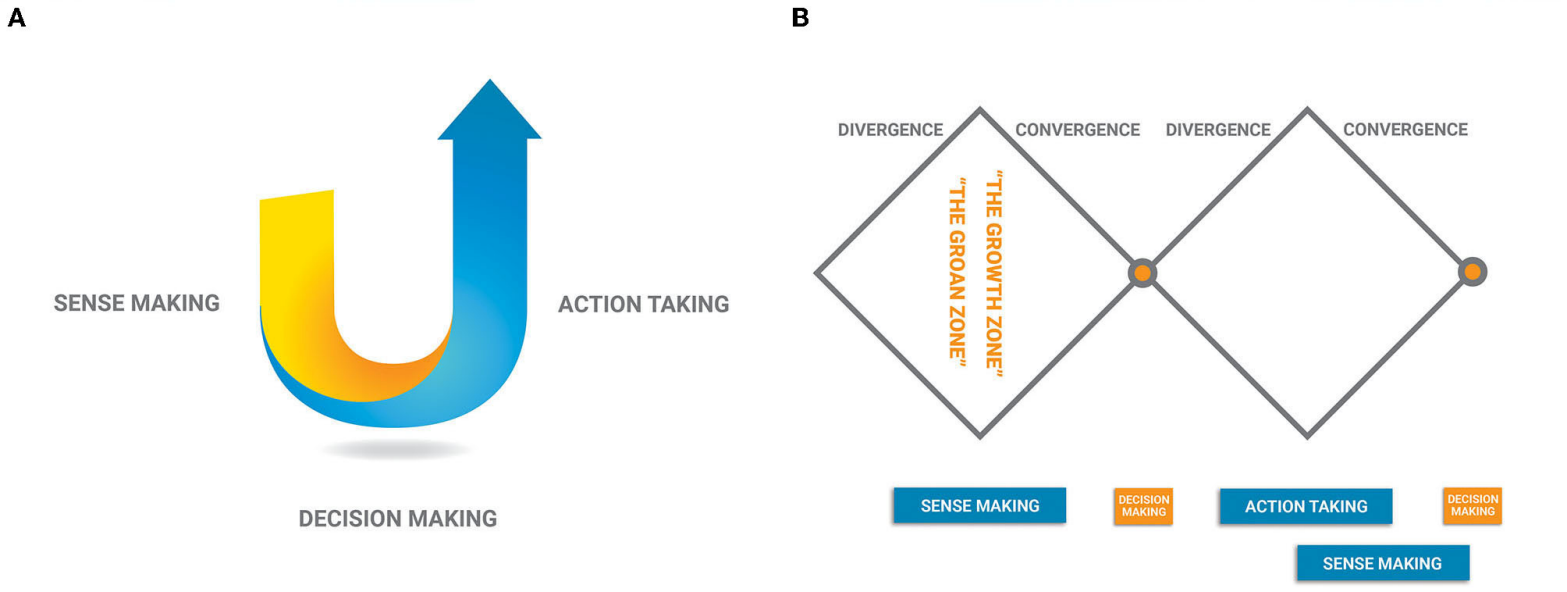
**(A)** The U framework. **(B)** The divergence-convergance framework.

Theory U (Figure 1A) is a meta leadership framework with wide applications in many contexts. In regards to collaborative leadership, the U movement captures the universal process of sense making, decision making and action taking: Going down the left side of the U is about sense making; the bottom of the U is about decision making; coming up the right side of the U is about action taking. In its original conception, going down the left side of U is termed as “observe, observe, observe”, the bottom of the U is dubbed as “retreat and reflect”, and coming up the right side of the U is called “act in an instant” (Scharmer, 2009).

The unique contribution of Theory U is the invitation to go deep down the left side of the U in the sensemaking process. The U shape indicates that the depth of sensemaking down the left side corresponds to the effectiveness of action taking up the right side. Sometimes when faced with a pressing challenge, the reactive parts of teams might want to take a short cut, to jump from the left side of the U directly to the right side of the U without going through a deep and inclusive sense making process. This shortcut often leads teams to “same old same old”, “business as usual” solutions. This is how solutions can become part of the next problem. This U movement invites teams to go deeper, beyond the kind of thinking that created the problem. When teams make sense of things differently, they make decisions differently and take actions differently.

The OODA loop, which stands for Observe, Orient, Decide and Act, coincides with the U movement. Developed during the Vietnam War to train the fighter jet pilots and popularized by the agile movement (Sutherland and Sutherland, 2014), the OODA loop also puts a strong emphasis on the sense making process. The first step to observe invites us to first move out of ourselves in order to see the whole picture; then to observe the response to the action from the system. The second step to “orient” expands the sense making process by incorporating a reflection on our mental model, the lens through which we see the world. It is highly likely that the lens itself might have been the problem. The invitation to observe and orient further illustrates the depth of sense making called for by going down the left side of U.

Theory U offers important insights for teams stuck in Pitfall #2 and #3 to engage in deep and inclusive sense making process.

### 3.2. The Divergence-Convergence Diamond

The Divergence-Convergence Diamond (Figure 1B) (Kaner, 2014) offers a second useful framework. We extend the original diamond to a “double diamond” to illustrate the dynamics of collaborative leadership. The sense making process goes through the first divergence-convergence diamond. At the beginning of the sense making process, team members express divergent views as a function of their diverse backgrounds. As these ideas interact, they merge, change, and morph into new ideas. At some point, ideas start to converge. However, teams can enter into a “groan zone” when they are unable to integrate the very divergent opinions. It can feel like indigestion - teams have not developed the capacity to integrate divergent information. The conflicting and connecting dynamics in the groan zone compel groups to engage heterogeneous perspectives, which is an essential step toward innovative solutions (Uhl-Bien, 2021). That is why “groan zone” is also referred to as “growth zone”. It is also possible that the pressure in the groan zone could be too much for teams to handle, and consequently break teams apart before a decision is reached, producing a suboptimal outcome.

Toward the end of the first diamond when there is enough consensus, teams make a decision and start the second divergence-convergence diamond with the action taking phase. As individual team members take actions, they make sense of the responses they are getting from their local contexts, the information they collect can be divergent from each other. The divergent information could in turn lead to the need to adapt the original decision, which enters the team into the first divergence-convergence diamond again.

At a micro-level, teams can travel through the divergence-convergence cycle over the course of a meeting where input is gathered, a decision is reached, and an action plan is made. At the macro-level, science teams can travel through a meta-cycle over the course of their five-year funding period, with several smaller divergence and convergence patterns embedded.

The Divergence-Convergence Diamond offers important insights for teams stuck in Pitfall #1 to move through sensemaking, reach a decision and start prototyping. Section 4.2 explores this further.

### 3.3. Strategic Doing

**Figure 2:**
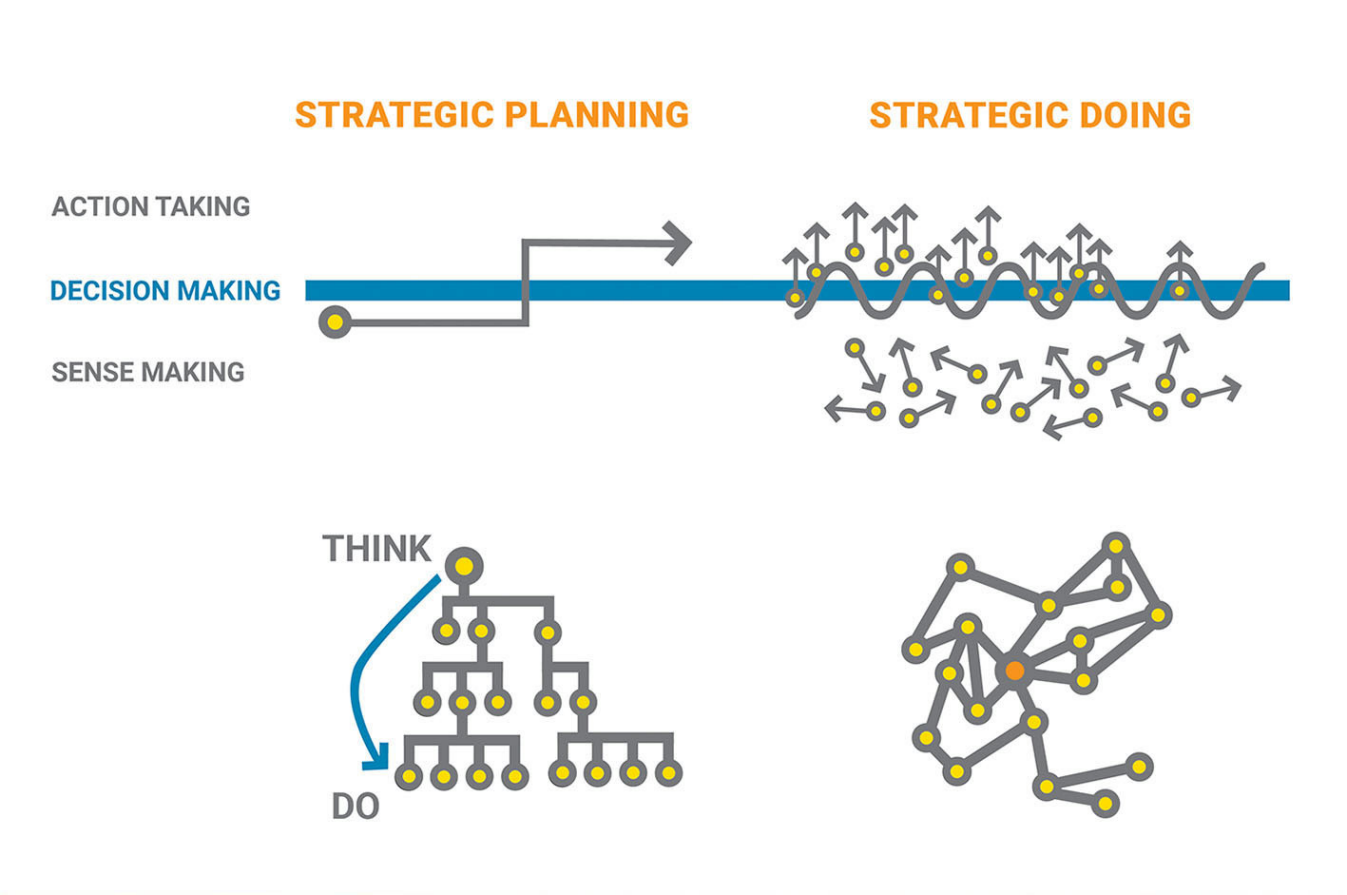
The strategic doing framework.

Strategic Doing (Figure 2) (Morrison et al., 2019) answers the two classic questions in strategic planning “where are we going” and “how will we get there” with a facilitated process guided by ten rules. The Strategic Doing (SD) process departs from traditional strategic planning in two main ways: speed of iteration, and integration of thinkers and doers.

Traditional strategic planning process takes a very long time in the realm of thinking. When a strategy is finally formulated, usually by the people at the top of hierarchy who are the designated “decision makers”, they “roll out” the strategy into the action taking realm, to people who are at the bottom of the hierarchy who are usually not involved in the sense making and decision making process. The approach has almost no learning loop among the sense making, decision making and action taking stages. In contrast, the Strategic Doing process assumes a network-based structure that prioritizes connectivity over hierarchy. All team members are involved in the sense making, decision making and action taking process, and travel through the process in fast iterations. Building on the context set by Rules 1–2, teams go through the sense making process that harmonizes divergence and gain enough coherence to make a decision (Rules 3–6 in SD). As they take actions, they designate time to incorporate their sense making to improve their decisions by asking questions such as: Does our proposed course forward still make sense? Are any course corrections needed in light of what we've learned over the last 30 days (Rules 7–10 in SD)?

In response to the three pitfalls, Strategic Doing helps teams travel through the cycles of planning, deciding and doing rapidly and embeds learning in the whole process. This approach helps teams to adapt to change, complexity, uncertainty and ambiguity.

## 4. Discussion

The three conceptual frameworks offer three powerful visuals to understand the collaborative leadership dynamics. For science teams navigating the dynamics, we offer the following practical suggestions.

### 4.1. Facilitate the process

Facilitation could help move teams through the process by applying methods specific to their challenges. For teams stuck in Pitfall #1 perpetual sense making loop, facilitation methods such as “The Debate” (Jiang, 2021) and the “Gradient of Agreement” (Kaner, 2014) could help move them toward a decision. On the other hand, for teams that have reached decisions prematurely (Pitfalls #2 and 3), facilitation methods such as 1–2-4-All, and Conversation Café (Lipmanowicz and McCandless, 2013) could help open up the space for divergence and creativity that feed into more effective decision making. By identifying where they are relative to the divergence-convergence diamond (Kaner, 2014), teams can apply effective facilitation methods.

### 4.2. Prototype the future

In complex adaptive systems, change is constant and unpredictable. This means that sense making is never exhaustive, decision making is never final and action taking needs constant course corrections. When teams prototype their ideas, they make sense of responses to their experiments and leverage the sensemaking to inform the next round of decision making and action taking. This prototyping approach is much more effective than staying in a conceptual sensemaking loop (Pitfall #1), which is nothing more than guesswork of unpredictable reactions to their proposed course forward.

The concept of “path finding projects” in Strategic Doing (Morrison et al., 2019) is an important example of prototyping the future. As teams engage in their path finder projects, they will generate more momentum toward the direction they started on, making course correction along the way, or determine it is not a viable path and seek other opportunities.

### 4.3. Dynamic roles and responsibilities

Sometimes science collaboration is mistakenly understood as “let's all do this together”, or “the more perspectives, the better”. It sometimes plays out as everyone plays a “consulted” role, but no one is “responsible” or “accountable” for anything. As a result, responsibilities fall through the crack and initiatives get stuck. Teams can gain more clarity about roles and responsibilities by coupling the dynamics of sense making, decision making and action taking with the RACI framework (Responsible, Accountable, Consulted and Informed) (Miranda and Watts, 2022).

The first step is to determine an *accountable* person: who is accountable for the deliverables? Who oversees the whole U process? This person needs to be a key stakeholder in the situation and has the authority to enlist their team members to engage in the sense making, decision making and action taking process. Without an accountable person for a specific objective, team members tend to default to the traditional hierarchy and look to senior scientists for solutions. But senior scientists are often over-stretched with other priorities, and in many cases, they are not the people closest to the situation. It is important to intentionally select the accountable person most appropriate for the situation.

The second step is to consider the roles needed in each of the three stages. In the sense making stage going down the left-hand side of the U, incorporating voices from those in *consulted* and *informed* roles could feed into better decisions. To reach a decision and move through the bottom of the U, teams need to bring coherence in the sense making process. It is therefore effective to keep the decision-making circle contained to those in *responsible* and *accountable* roles. Action taking up the right-hand side of the U is about assigning specific responsibilities to specific individuals, so it is advisable to focus on the *responsible* roles. When the need to make course corrections arises, those in *consulted* roles could be brought in again to help the *accountable* and the *responsible* make sense and go down the left-hand side of the U again.

For teams stuck in “analysis paralysis” (Pitfall #1), it might help to close their ears to those in the *consulted* and *informed* roles for a while and allow those *accountable* to make a decision and for those *responsible* to move forward with actions. On the other hand, for teams needing adaptations to their plans (Pitfall #3), it might help to do just the opposite. The *accountable* and *responsible* could gain valuable insights by inviting those in *consulted* and *informed* roles to make sense of the changed situations again. Discernment is key in dynamically including the different roles in the three distinct stages.

## 5. Conclusions

For cross-disciplinary science teams navigating complexity, this perspective article offers three practical frameworks to operationalize collaborative leadership as iterative cycles of distributed sense making, decision making and action taking. This is a useful starting point. Some further questions for leaders, scholars and practitioners interested in enabling cross-disciplinary science teams include: how might we better facilitate smooth transitions from sense making to decision making to action taking? How might we apply a dynamic view toward roles and responsibilities in relationship to the three stages? How might a prototyping spirit be fostered in the sometimes risk-averse science culture? What are other ways of operationalizing collaborative leadership?

## Data availability statement

The original contributions presented in the study are included in the article/supplementary material, further inquiries can be directed to the corresponding author.

## Author contributions

The author confirms being the sole contributor of this work and has approved it for publication.
